# Homicide–suicide and the role of mental disorder: a national consecutive case series

**DOI:** 10.1007/s00127-016-1209-4

**Published:** 2016-04-16

**Authors:** Sandra Flynn, Linda Gask, Louis Appleby, Jenny Shaw

**Affiliations:** The National Confidential Inquiry into Suicide and Homicide by People with Mental Illness, Centre for Mental Health and Safety, Institute of Brain Behaviour and Mental Health, University of Manchester, Jean McFarlane Building, Oxford Road, Manchester, M13 9PL UK; Institute of Population Health, University of Manchester, Williamson Building, Oxford Road, Manchester, M13 9PL UK

**Keywords:** Homicide, Suicide, Homicide–suicide, Family violence, Mental illness, Depression

## Abstract

**Purpose:**

There is a lack of robust empirical research examining mental disorder and homicide–suicide. Primary care medical records are seldom used in homicide–suicide research. The aims of this study were to describe the characteristics of offenders and victims; determine the prevalence of mental disorder and contact with mental health services and examine adverse events prior to the offence.

**Methods:**

This was a mixed-methods study based on a consecutive case series of offences in England and Wales occurring between 2006 and 2008. 60 homicide–suicides were recorded. Data sources included coroner’s records, police files, General Practice (GP) and specialist mental health records, and newspaper articles.

**Results:**

The results show that most victims were spouse/partners and/or children. Most perpetrators were male (88 %) and most victims were female (77 %). The incidents were commonly preceded by relationship breakdown and separation. 62 % had mental health problems. A quarter visited a GP for emotional distress within a month of the incident. Few had been in recent contact with mental health services before the incident (12 %). Self-harm (26 %) and domestic violence (39 %) were common.

**Conclusion:**

In conclusion, GPs cannot be expected to prevent homicide–suicide directly, but they can reduce risk generally, via the treatment of depression and recognising the risks associated with domestic violence.

## Introduction

Homicide–suicide is where an individual kills another person and then takes their own life. In the majority of cases the suicide occurs immediately. In our definition we have also included those who died within 3 days of the homicide, and cases where the offender was fatally injured at the time of the homicide, but died more than 3 days later. This is consistent with previous studies [1–3]. Over recent years, homicide–suicide has received increased academic attention, both empirically and theoretically, which has advanced our understanding of the phenomena. These acts are mostly carried out by men, the victims are most often female intimate partners or close family members, multiple victims are also common.

Explanations why people commit these acts have been proposed and classified in numerous typologies. Motivations include jealousy and revenge following real or perceived infidelity and relationship breakdown, altruism or mercy killing, financial problems and mental disorder [1, 4, 5]. However, these typologies have their limitations and do not comprehensively capture the complexities of homicide–suicide. Reliable information on the role of mental disorder in these cases is lacking. In a review of the literature spanning 60 years, Roma et al. (2012) found 30 studies presenting clinical findings, 20 reporting depression and 11 reporting psychosis [6]. Of these studies, the definition of mental disorder used have resulted in a wide variation in the prevalence rates reported (18–75 %) [7, 8]. Definitions of mental illness have included serious mental illness that required hospitalisation [7]; any treatment received from specialist mental health care services [2]; recorded history of mental illness documented by the police and/or medical examiners (National Violent Death Reporting System US) [9]; or having a post mortem diagnoses derived from medical records and/or informant descriptions of the deceased’s mental state (psychological autopsy) [8]. Whilst these studies have provided valuable clinical insight, they are often based on subgroups such as filicide-suicide [10]; older couples [11]; or intimate-partner homicide–suicide [12–15]. Furthermore, the findings are commonly based on small regional samples [16–18]. Therefore due to the difficulties with data collection, national clinical studies of homicide–suicide are rare. Furthermore, mental disorder diagnosed by primary care and recorded in general practice medical records are seldom accessed.

In this study, we aimed to further our understanding of homicide–suicide by firstly describing the characteristics of offenders and victims. Secondly, we aimed to examine the clinical antecedents of these incidents (i.e. history of mental disorder, contact with mental health services, diagnosis, medication prescribed). Thirdly we aimed to examine adverse events prior to the offence (i.e. relationship breakdown and child custody disputes).

The low incident rate of homicide–suicide in England and Wales of 0.05 per 100,000 population (approximately 23 incidents per year) enabled a detailed exploration of the complex aetiology of these cases. Therefore, this is the first study to complete an in-depth examination using multiple data sources on a national consecutive case series of homicide–suicide.

## Materials and methods

### Research design

A mixed-method design was used to examine data from a national consecutive case series of homicide–suicides in England and Wales between 1st January 2006 and 31st December 2008. The case series was accessed via the National Confidential Inquiry into Suicide and Homicide by People with Mental Illness (NCISH) [3]. NCISH collate and maintain clinical data on homicide and suicide not replicated by any other national or international research group or organisation. Access to this data makes these finding unique. Additional information for both the qualitative and quantitative phases of the study were collected and analysed concurrently (i.e. both methods were used to explore the same research questions at the same time) [19].

### Sample: selection of participants

A total of 83 people were initially suspected of carrying out a homicide–suicide over the study period. Twenty-three were excluded for reasons including; the perpetrator took their own life more than 3 days after the homicide (18 offenders); in 4 cases the coroner did not return an unlawful killing or suicide verdict; and in 1 case the inquest had not taken place at the time of analysis, and therefore a verdict had not been determined. The final sample consisted of 60 cases.

### Data collection

Cases were identified using the existing NCISH homicide–suicide database. A detailed description of methodology for recording homicide–suicide incidents has been described previously [2]. To briefly summarise, notification of homicide–suicide incidents was received from the Home Office Statistics Unit of Home Office Science in most cases (92 %). The remaining cases not recorded on the homicide index were notified to NCISH via individual police forces. Data held on the NCISH database included demographic characteristics of the offender and victim, offence details and information regarding diagnosis and contact with mental health services. Once the offender had been identified, additional information was requested and obtained from coroners’ files and police records (82 % of cases), General Practice (GP) medical records (88 % of cases) and newspaper articles (90 % of cases). The files contained antecedent information describing the events, circumstances and mental state of the offender leading up to the incidents which were specified in witness statements by friends and family members. GP medical records were examined to determine recent attendance, history of mental disorder, recent symptoms and medication prescribed. Triangulation of these data sources was undertaken to ensure the validity of responses. In instances of non-convergence between data sources, inconsistencies were reconciled by reviewing the evidence from all sources, discussing this amongst the authors and determining the most reasonable finding.

### Quantitative data analysis

Data analysis was conducted using Stata version 11. Rates were calculated using ONS mid-year population estimates. Results were reported using 95 % confidence intervals. If an item of information was not known for a case, the case was removed from the analysis of that item; the denominator in all estimates was the number of valid cases for each item and indicates the number of missing cases per item.

### Qualitative data analysis

Documentary analysis was undertaken in accordance with Hodder [20]. The content of the documents such as the coroner’s report, police investigation reports and witness statements were explored using framework analysis [21]. This systematic and comprehensive approach was undertaken in 5 key stages; familiarisation with the content of the documents; identifying an initial thematic framework; indexing or coding the data; charting the salient text to the framework; mapping themes and interpreting the meaning of these themes. An iterative approach was applied whereby the themes were repeatedly refined until saturation point was reached and no new themes emerged.

This method was preferred to the alternative of mapping to existing typologies [1, 5] as it enabled the generation of themes from our unique national consecutive case-series. Coding was undertaken by SF and the thematic framework was refined by SF, LG and JS during the iterative process.

## Results

### The characteristics of perpetrators and victims

During the 3 year study period 60 people, 4 % of offenders took their own life after committing homicide, a rate of 0.04 per 100,000 population. The characteristics of offenders and victims are presented in Table 1. Most of the offenders were male. The median age was 44. Half were married or cohabiting. The majority were white. Almost a third had a conviction for violence, including 3 offenders who had previously been convicted of homicide. The victims of the previous homicides were a former partner in 2 cases and an acquaintance in the remaining case. Over a third of offenders were found to have previously committed domestic violence. There were 70 victims in total, the majority of whom were female. The median age of the victims was 38. Nearly two-thirds of victims were the offender's spouse/partner (current or ex), of these 19 (45 %) had been in an intimate relationship for over 20 years. Twenty (29 %) children were killed by a parent (filicide) and 2 perpetrators killed a spouse/partner (current or ex) and children (familicide). In total, 6 offenders killed more than 1 victim. The highest number of victims killed in single incident was 5.

**Table 1:** Characteristics of homicide–suicide

Offender characteristics	*N* = 60	Valid (%)	95 % CI
Demographic characteristics			
Age of offender: median (range)	44	(18–85)	
Male offender	53	88 %	80–97
Married/cohabiting	32	53 %	40–66
Married but separated	7	12 %	3–20
Unemployed	16	28 %	16–40
Living alone	9	15 %	9–25
Black or minority ethnic group	17	29 %	17–41
Born outside the UK	14	24 %	13–35
Behavioural features			
History of alcohol misuse	15	28 %	16–41
History of drug misuse	13	23 %	12–35
Any previous conviction	27	45 %	32–58
Previous conviction for violence	18	30 %	18–42
Previously committed domestic violence	22	39 %	26–52
History of self-harm/attempted suicide	14	26 %	14–38
Previously bereaved by suicide	5	9 %	2–17
Offence characteristics			
Suicide occurred <24 h after homicide	51	85 %	76–94
Homicide occurred in shared home	31	52 %	39–65
Suicide occurred in shared home	25	42 %	29–55
Method of homicide			
Sharp instrument	22	37 %	24–49
Asphyxia (strangulation/suffocation)	18	30 %	18–42
Firearms	6	10 %	2–18
Method of suicide			
Hanging	20	33 %	21–45
Cutting/stabbing	10	17 %	7–26
Firearms	5	8 %	1–15
Self-poisoning	5	8 %	1–15
Drowning	5	8 %	1–15
Same method used in homicide and suicide	24	40 %	28–52

Victim characteristics	*N* = 70	Valid (%)	95 % CI
Demographic characteristics			
Age of victim: median (range)	38	(1–85)	
Female victim	54	77 %	67–87
Unemployed	11	16 %	7–26
Ethnic minority	15	21 %	10–30
Relationship of victim to perpetrator			
Spouse/partner (current or ex)	45	64 %	53–76
Child or stepchild	20	29 %	18–39
Multiple victims killed in an incident	6	10 %	2–18
Victims were spouse/partner and children (familicide)	2	3 %	1–8

### Method of homicide and suicide

In the majority of incidents the offender took his or her own life within 24 h of committing homicide and this occurred most often in home shared by the offender and victim. The most common method of homicide was by sharp instrument, and hanging was the most frequently used method of suicide. The same method was used in both homicide and suicide in 24 (40 %) cases (Table 1).

### Adverse events prior to the homicide–suicide

The most common circumstances leading to the individual’s emotional distress was the loss of a close personal relationship either through imminent separation or divorce; or a significant change in the relationship due to the victim’s ill health (e.g. dementia). Other adverse events included problems with children (child custody/access problems or the perpetrator’s belief that they were failing as a parent); financial problems; and adjusting to a new social situation (either because they were a recent immigrant or a recently released prisoner). Most offenders had previously exhibited difficulty coping with emotional distress, resulted in violence and aggression or self-harm. Five (9 %) had previously been bereaved by the suicide of a family member or close friend. In 1 case the offender experienced the loss within 2 weeks of the homicide–suicide, in the remaining cases the deaths occurred more than 10 years before the homicide–suicide.

### History of mental disorder prior to the offence

Fifty-six (93 %) offenders were registered with a GP practice. Data from GP medical records were obtained on 53 (88 %) offenders. The following analysis is based on those 53 cases on whom data were available (Table 2). Thirty-three (62 %) had previously been diagnosed with a mental disorder. The most common diagnosis was depression, psychosis was rare, and none of the offenders had been diagnosed with personality disorder. Nearly a third had been prescribed psychotropic medication at the time of the homicide–suicide, mostly antidepressants. A quarter had previously attempted suicide between 1 and 4 times. Overall, suicidal ideation was noted by a GP in 7 cases (14 %). Three quarters of the perpetrators had attended their GP within 12 months of the offence, 40 % within a month. A third of offenders were recorded as having discussed psychological problems with their GP within a year of the offence, a quarter within a month.

**Table 2:** Mental disorder recorded in medical records

Clinical characteristics	*N* = 53	Valid (%)	95 % CI
Evidence of mental illness			
Diagnosis and prior treatment for mental illness (lifetime)	33	62	49–76
Primary diagnosis			
Depressive disorder	28	53	39–67
(Situational depression)	(6)	12	4–23
Other diagnosis (e.g. stress/anxiety)	2	4	0–9
Schizophrenia and other delusional disorders	1	2	0–6
Alcohol dependence	1	2	0–6
Drug dependence	1	2	0–6
No mental illness	20	38	24–51
Medication			
Psychotropic medication prescribed at the time of the offence	14	30	17–44
Contact with GP services			
Recent contact with GP			
Contact within 12 months of the offence	41	77	66–89
Contact within 1 month of the offence	21	40	26–53
Contact with for psychological problems			
Contact within 12 months of the offence	22	42	28–55
Contact <1 month prior	15	28	16–41

Contact with mental health services	*N* = 60	Valid (%)	95 % CI
Any previous contact with MH services	14	23	13–34
Contact within 12 months of the offence	7	12	4–20
Contact <1 month prior	4	7	0–13

A response to our inquiry regarding contact with specialist mental health services was received on all 60 offenders. The majority (46, 77 %) had no previous contact with mental health services. Of the fourteen (23 %) who had a history of contact 7 were former patients and 7 had been under mental health care within 12 months of the offence, most commonly for depression. Ten (19 %) had previously been admitted as an in-patient (Table 2).

### Classifying homicide–suicide

The homicide–suicide offenders diverge into two groups, firstly offenders with a history of depression and secondly offenders with a history of perpetrating domestic violence. Of the first group with depression (*n* = 28), 16 (57 %) had consulted a GP a month before the incident, mostly to discuss psychological problems. Eleven (41 %) had previously self-harmed/attempted suicide. Most depressed patients had been prescribed psychotropic medication at the time of the incident (23, 85 %). The most common adverse incident reported by witnesses in this group (aside from mental illness) was separation from their partner (12, 46 %). The second group identified were offenders with a history of committing domestic violence (22, 39 %). Of these 22, 14 (64 %) had previous convictions, this included violent offences in 11 (50 %). A history of substance misuse was common (10/19, 53 %). Separation from a partner was the most common adverse event to occur prior to the homicide (11/21, 52 %). There was an overlap of 7 offenders who had both a history of mental illness and of committing domestic violence.

## Discussion

This is the only study to our knowledge to have undertaken an in-depth examination of homicide–suicide and mental disorder on a national case-series using data from primary care and specialist mental health services. The use of information from a variety of robust data sources and utilising a mixed method research design makes this study unique. We found that perpetrators of homicide–suicide in this study were commonly middle-aged white males, who recently experienced a relationship breakdown. Mental disorder, particularly depression was common. Almost two-thirds of those on whom medical records were obtained had previously been diagnosed with a mental disorder. Although the overall proportion with mental disorder was high, the majority did not have serious mental illness requiring care under specialist mental health services. This is consistent with NCISH data on convicted homicide offenders (10 %) but was lower than the proportion of people who died by suicide in recent contact with mental health services in England (28 %) [3]. It was more common for the offender to be seen by primary care services. Contact with GP services within a year of the incident (77 %) and within a month (40 %) was remarkably similar to the proportion of service contact in deaths by suicide in the general population, 77 % and 45 % respectively [22]. Almost a third had been prescribed antidepressants at the time of the offence but we were unable to confirm if medication was being taken as instructed. Non-adherence is estimated to occur in 50 % of patients prescribed antidepressants, which can lead to an increased risk of adverse symptoms [23, 24].

Domestic violence was found to be an important feature of these cases, with over a third of offenders having previously assaulted a partner, similar to previous studies [25, 26]. However, few of these offenders had a history of mental disorder. This contrasts with findings from a US psychological autopsy study that found most offenders had a history of domestic violence as well as depression [27]. We recognise that personality disorder was likely to be under reported in this case series, as the diagnosis was not recorded in either the primary care or mental health service medical records. Knoll and Hatters-Friedman (2015) reported 17 % of perpetrators had antisocial personality disorder, but their sample size was limited (*n* = 18) [27]. It is challenging to identify personality traits retrospectively in a deceased offender. A potential solution would be use document-derived assessments instruments such as the PAS-DOC [28] to examine the possible association between personality disorder and homicide–suicide.

### Recognising and treating patients at risk

We found the proportion of people who committed homicide–suicide and had a history of mental illness was consistent with previous studies using a broadly similar methodological approach [29, 30]. However, the proportion was much higher than reported in US samples [9, 25]. The literature shows that these acts are commonly carried out by middle-age men, which supports the evidence that this is an emerging high risk group for suicide [31]. Personal loss through relationship breakdown at this stage in life has been shown to be an important trigger in these incidents [32], but the causes of homicide–suicide extend beyond emotional stress arising from relationship difficulties (which are not uncommon problems). The key factor associated with homicide–suicides is the individual’s lack of resilience and inability to cope with stressful events, evidenced by their reaction to previous similar experiences, either responding violently towards themselves or other people. Despite this recorded history of high risk behaviour and emotional instability these factors were not commonly explored by GPs through routine enquiry. Previous research has highlighted a reluctance to discuss emotional problems with patients in fear of opening ‘Pandora’s box’ [33]. However, such discussions are necessary to differentiate between emotional distress and a diagnosis of mental disorder requiring referral to specialist mental health services.

### Limitations

There are inherent difficulties in using retrospective data and documents originally generated for non-research purposes that can introduce bias. However, given the low base rate of these events and the fact that both parties involved are deceased, data are often limited and difficult to obtain. We carried out a retrospective analysis of medical records to determine whether the offender had been diagnosed with any mental disorder. The shortcomings identified in using diagnoses recorded in official documents to measure mental illness were; (1) the true prevalence of mental illness may be underestimated as medical help is not always sought by people experiencing mental health problems; (2) the reliability of the recorded diagnoses has not been verified and there are likely to be inconsistencies between cases; (3) it is not known whether the diagnostic data in this study accurately reflects actual symptom-based diagnoses in a reliable and valid way.

There are also difficulties in determine a person’s mental state at the time of offence based on descriptions provided in documents. This can only be achieved reliably through interviews and a psychiatric assessment of the individual following the offence, which is not possible with homicide–suicide offenders. In addition, due to the methodology used in this study we cannot state that there was a causal relationship between mental illness and the offence. We have therefore referred to the individual’s history of mental illness or depression, and acknowledge that although a person may have a history of mental illness, they may not have had active symptoms at the time of offence, or impaired functioning. Despite the shortcomings, we consider this to be the most robust method available for defining mental illness in this population. Corroborating the findings from numerous sources of data through triangulation provided validity and rigour to the findings and helped to enhance our understanding of these events.

### Clinical implications

It would be unrealistic to expect GPs or psychiatrists to prevent homicide–suicide directly, as these incidents are relatively rare and few clinicians will ever experience this phenomenon. There are however, improvements in service delivery that could help to reduce the number of incidents. For example the recognition and better treatment of mental disorder, particularly depression in primary care is achievable. Previous research has found that people with serious mental health problems, particularly major depression, are more likely to visit their GP, which demonstrates help seeking behaviour and a willingness to engage [34, 35]. It is of course more difficult to recommend preventative action for people not under the care of health services. Our findings suggest that only a quarter of the offenders who experienced emotional distress contacted their GP for psychological support in the weeks before the incident, the majority did not. Previous research has shown that people may be reluctant to engage with services due to self-stigma (personal views about mental illness) [36] and the public stigma (society’s perception of mental illness) [37]. Therefore, it is important to promote campaigns to reduce stigma and to raise awareness of the wide range of support available and how this can be accessed. Initiatives in the voluntary sector such as State of Mind are a good example of how this can been achieved. The organisation raises awareness of mental health issues through sport, working to improve the mental health of professional and amateur rugby league players, the wider community and students at local colleges [38].

Secondly, our data has shown a small proportion of homicide–suicide offenders had mental illness and a history of committing intimate partner violence. A recent meta-analysis has shown an association between domestic violence perpetration and mental illness [39]. Men with depression were shown to have an almost 3-fold increase risk of committing intimate partner violence. However, the evidence base is currently insufficient, and more research is required to identify risk factors which will inform prevention strategies. Increasing our knowledge of risk factors in this population will help to reduce the risk of future incidents.

### Ethical considerations

The study received MREC approval on 9th April 2008 and is registered under the Data Protection Act. Exemption under section 251 of the NHS Act 2006 (formerly section 60 of the Health and Social Care Act 2001) was obtained enabling access to confidential and identifiable information without informed consent in the interest of improving patient care (approved 23rd October 2008). Research governance approval was sought from 49 Primary Care Trusts in England and Wales. The study was registered under the Data Protection Act (1998). The authors assert that all procedures contributing to this work comply with the ethical standards of the relevant national and institutional committees on human experimentation and with the Helsinki Declaration of 1975, as revised in 2008.
